# When Nothing Goes Right: Risk Factors and Biomarkers of Right Heart Failure after Left Ventricular Assist Device Implantation

**DOI:** 10.3390/life12030459

**Published:** 2022-03-20

**Authors:** Thomas Schlöglhofer, Franziska Wittmann, Robert Paus, Julia Riebandt, Anne-Kristin Schaefer, Philipp Angleitner, Marcus Granegger, Philipp Aigner, Dominik Wiedemann, Günther Laufer, Heinrich Schima, Daniel Zimpfer

**Affiliations:** 1 Department of Cardiac Surgery, Medical University of Vienna, 1090 Vienna, Austria; franziska.wittmann@meduniwien.ac.at (F.W.); n1613747@students.meduniwien.ac.at (R.P.); julia.riebandt@meduniwien.ac.at (J.R.); anne-kristin.schaefer@meduniwien.ac.at (A.-K.S.); philipp.angleitner@meduniwien.ac.at (P.A.); marcus.granegger@meduniwien.ac.at (M.G.); dominik.wiedemann@meduniwien.ac.at (D.W.); guenther.laufer@meduniwien.ac.at (G.L.); heinrich.schima@meduniwien.ac.at (H.S.); daniel.zimpfer@meduniwien.ac.at (D.Z.); 2 Ludwig Boltzmann Institute for Cardiovascular Research, 1020 Vienna, Austria; philipp.aigner@meduniwien.ac.at; 3 Center for Medical Physics and Biomedical Engineering, Medical University of Vienna, 1090 Vienna, Austria

**Keywords:** ventricular assist device, mechanical circulatory support, right heart failure, risk factor

## Abstract

Right heart failure (RHF) is a severe complication after left ventricular assist device (LVAD) implantation. The aim of this study was to analyze the incidence, risk factors, and biomarkers for late RHF including the possible superiority of the device and implantation method. This retrospective, single-center study included patients who underwent LVAD implantation between 2014 and 2018. Primary outcome was freedom from RHF over one-year after LVAD implantation; secondary outcomes included pre- and postoperative risk factors and biomarkers for RHF. Of the 145 consecutive patients (HeartMate 3/HVAD: n = 70/75; female: 13.8%), thirty-one patients (21.4%) suffered RHF after a mean LVAD support of median (IQR) 105 (118) days. LVAD implantation method (less invasive: 46.7% vs. 35.1%, *p* = 0.29) did not differ significantly in patients with or without RHF, whereas the incidence of RHF was lower in HeartMate 3 vs. HVAD patients (12.9% vs. 29.3%, *p* = 0.016). Multivariate Cox proportional hazard analysis identified HVAD (HR 4.61, 95% CI 1.12–18.98; *p* = 0.03), early post-op heart rate (HR 0.96, 95% CI 0.93–0.99; *p* = 0.02), and central venous pressure (CVP) (HR 1.21, 95% CI 1.05–1.39; *p* = 0.01) as independent risk factors for RHF, but no association of RHF with increased all-cause mortality (HR 1.00, 95% CI 0.99–1.01; *p* = 0.50) was found. To conclude, HVAD use, lower heart rate, and higher CVP early post-op were independent risk factors for RHF following LVAD implantation.

## 1. Introduction

Terminal heart failure (HF) remains one of the most important health problems of our time, both socially and economically. In addition to optimal medical therapy or heart transplantation, implantation of a left ventricular assist device (LVAD) represents standard of care for end-stage HF patients [1]. The modus operandi for LVAD therapy is to support the left ventricle by bypassing blood flow through the pump directly into the systemic circulation [2]. Obviously, LVAD therapy mainly treats the insufficiency of the left ventricle. In patients with failure of the left ventricle, the right ventricle (RV) is always also affected to a varying extent. Implantation of a LVAD can lead to either changes in RV hemodynamics by decreasing RV afterload, but also increasing venous return and thus filling pressure [3], or disruption of RV mechanics by a leftward septal shift caused by left ventricular decompression, all of which can lead to RHF [4]. Therefore, patient evaluation before LVAD implantation must carefully consider right ventricular function, risk for subsequent RHF, and the need for implantation of a temporary (tempRVAD) or permanent right ventricular assist device (RVAD). In the majority of HF patients with mild-to-moderate RHF, LVAD implantation results in the stabilization of RV function [5]. However, these patients remain at risk for developing late RHF during LVAD support, which is an important concern, particularly in destination therapy (DT) but also in bridge to transplantation (BTT) or bridge to candidacy (BTC) patients with a long estimated duration of LVAD support. Many models of RHF post-LVAD implantation use preoperative risk factors [6] such as tricuspid regurgitation or elevated central venous pressure (CVP) [7,8], while others describe biomarkers of intrinsic RV function such as elevated blood urea levels as independent risk factors [9]. On the other hand, a less invasive surgical implantation strategy has been suggested to have a protective effect on the development of RHF after LVAD implantation [10]. Recent studies have suggested the superiority of the HeartMate 3 (HM3) (Abbott Inc., Chicago, IL, USA) over the HVAD (Medtronic Inc., Minneapolis, MN, USA) device in terms of the occurrence of thromboembolic events [11,12,13,14]; however, other analyses have failed to identify substantial differences [15]. A possible superiority of the HM3 vs. HVAD device in terms of the development of RHF has not been investigated. Therefore, the aim of this study was to analyze the incidence, risk factors, and biomarkers for RHF over time after LVAD implantation including the possible superiority of the device and implantation method to facilitate future pre- and perioperative assessment of the risk of subsequent RHF.

## 2. Materials and Methods

The study was approved by the Ethics Committee of the Medical University of Vienna (EK Nr: 1769/2018).

### 2.1. Study Population

**Figure 1:**
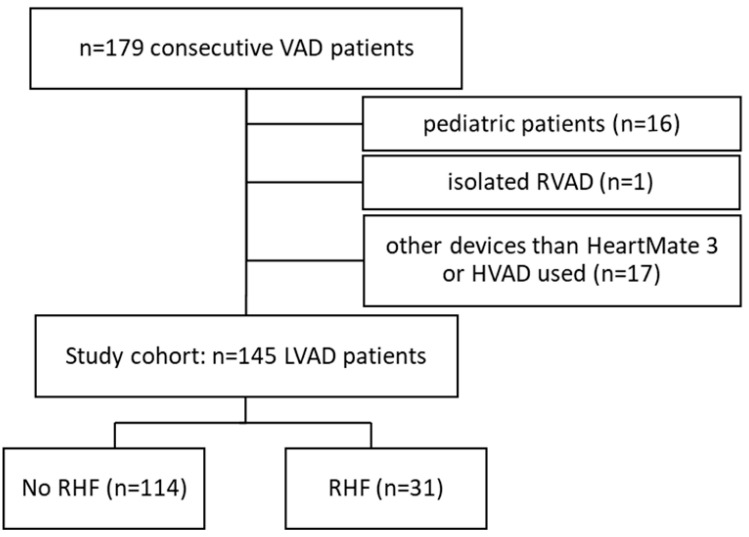
Patient flow chart. Of n = 179 consecutive VAD patients, n = 145 met all inclusion criteria and were included in the study cohort.

This retrospective, single-center study included n = 179 consecutive patients who underwent LVAD implantation between 2014 and 2018. We excluded patients <18 years of age (n = 16), patients with primary devices (isolated right ventricular support) other than LVAD (n = 1), and devices other than HM3 or HVAD (n = 17) (Figure 1). Freedom from RHF and clinical outcomes were followed for one-year after implantation.

### 2.2. Definition of RHF and Variables Evaluated

In accordance with the Interagency Registry for Mechanical Circulatory Support (INTERMACS) adverse event definitions version 5.0 [16], RHF was defined as the post-operative finding of both elevated CVP (>16 mmHg) and clinical findings of peripheral edema or laboratory evidence of worsening hepatic (total bilirubin > 2.0 mg/dL) or renal dysfunction (creatinine > 2.0 mg/dL); the need for inhaled nitric oxide for ≥48 h or intravenous inotrope therapy beyond post-op day 7 and/or tempRVAD or RVAD implantation in a second procedure after the initial LVAD implantation [17]. TempRVAD implantation at the time of initial LVAD implantation is an institutional approach for preoperatively impaired RV function to prevent postoperative RHF and to ensure adequate end-organ perfusion. Therefore, these patients were not included in the late RHF cohort as such.

Clinically relevant data were collected from the study cohort, ≤24 h before, early post-op (<4 h post implantation), and within a 30-day period to determine the clinical and hemodynamic predictors of RVF after LVAD implantation. Clinical variables obtained before LVAD implantation included demographics, comorbidities, medication (beta blocker therapy), and need for life support including mechanical ventilation or extracorporeal tempRVAD support. Intraoperative data included device type and pump parameters, implantation method (full sternotomy vs. less-invasive), type of bypass support, and concomitant procedures (tempRVAD or valve surgery). Pre- and postoperative laboratory data included a complete blood count, liver enzymes, lactate dehydrogenase (LDH), pro-brain natriuretic peptide (proBNP), albumin, and creatine kinase. Hemodynamic variables included CVP, pulmonary capillary wedge pressures, systolic pulmonary artery pressure, mean arterial blood pressure (MAP), cardiac output, and heart rate.

During the one-year follow-up period, regular outpatient follow-up visits were performed at one, three, six, and 12 months, during which laboratory and noninvasive hemodynamic parameters, medication, and pump parameters were screened for RHF. If the patient’s general condition worsened or if low flow alarms occurred, echocardiography was performed.

### 2.3. Study Design

The primary outcome in this study was freedom from RHF one-year after LVAD implantation; secondary outcomes included pre- and postoperative risk factors and biomarkers for the development of RHF and possible device superiority (HM3 vs. HVAD) and implantation method.

### 2.4. Statistical Analysis

Normally distributed continuous variables are presented as mean ± standard deviation, variables with non-normal distribution are depicted by median (interquartile range). Categorical variables are described as number (percentage). Normal distribution of continuous variables was assessed by the Shapiro–Wilk test. Fisher’s exact test was used to assess for statistical significance of categorical variables, Student’s *t*-test or Mann–Whitney U test for continuous variables. Time-to-event analysis was performed by using Kaplan–Meier curves with *p*-values reported using the log-rank test. Cox proportional hazard models were used to determine factors associated with one-year freedom from RHF and survival with RHF as the time-varying predictor. Variables with *p* < 0.2 in the univariate analysis entered the multivariate analysis; individuals with missing data in any covariate were excluded. Patient follow-up was censored when patients underwent heart transplantation, device explantation, or expired. Statistical analyses were performed with the use of SPSS for Windows Release 26.0.0 (IBM, Armonk, NY, USA). Statistical significance was set at *p* < 0.05.

## 3. Results

### 3.1. Patient Characteristics

Baseline demographics and comorbidities of the study cohort (n = 145), stratified by patients with and without RHF, are summarized in Table 1. The following two LVAD device types were used: 70 (48.3%) HM3 and 75 (51.7%) HVAD. A total of 21.0% received a VAD as BTT, 33.5% as DT, 44.8% as BTC, and 0.7% as bridge to recovery. Mean age of the patients was 60.1 ± 11.2 years, body mass index (BMI) was 27.3 ± 5.0 kg/m^2^, and 13.8% were female.

### 3.2. Incidence and Treatment of RHF

**Figure 2:**
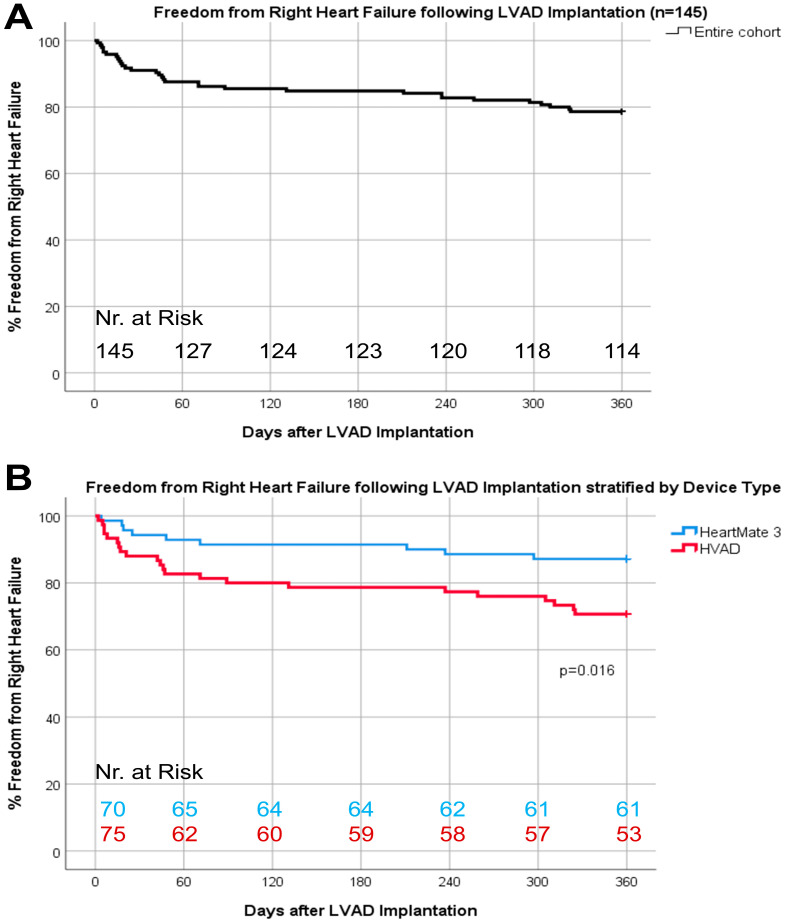
Baseline demographics including intraoperative variables, comorbidities, and preoperative laboratory and hemodynamic parameters of freedom from right heart failure one-year following LVAD implantation (**A**) and stratified by device type (**B**).

Thirty-one patients (21.4%) experienced RHF after a median LVAD support of 105 (118) days (Figure 2A) and 16.1% of these patients had at least one recurrent RHF event. No patient was lost to follow-up. The incidence of RHF was lower in HM3 vs. HVAD patients (12.9% vs. 29.3%, *p* = 0.016, Figure 2B) without differences in recurrent RHF (1.5% vs. 5.3%, *p* = 0.37). The majority of patients with RHF (n = 31) were treated with an increase in diuretic therapy (48.4%), intravenous inotropic therapy (45.2%); one patient (3.2%) received inhaled nitric oxide for ≥48 h; and another (3.2%) required tempRVAD implantation.

### 3.3. Risk Factors and Biomarkers for RHF and Impact on Survival

Less invasive LVAD implantation method (RHF: 46.7% vs. no RHF: 35.1%, *p* = 0.29) or concomitant use of a tempRVAD (RHF: 41.9% vs. no RHF: 28.1%, *p* = 0.19) did not differ significantly in patients with or without RHF (see Table 1). Table 2 shows the laboratory and hemodynamic variables assessed early post-op and 30-days after LVAD implantation. Univariable Cox proportional cause-specific hazards regression analysis (Appendix A Table A1) showed that HVAD use, higher CVP and lower heart rate early post-op period, and lower hemoglobin and platelets or elevated leukocytes, total bilirubin, and CRP 30 days after implantation were associated with significantly increased risk of RHF one-year following LVAD implantation. Based on the results of the univariable analysis, the following variables were included in the baseline multivariable model: beta blocker use, hemoglobin and CK preoperative; concomitant tempRVAD support and LVAD device type; heart rate, CVP early post-op; and thrombocyctes, leukocytes, CRP, total bilirubin, and albumin 30-days post LVAD implantation.

Multivariate Cox proportional hazard analysis identified the HVAD device (HR 4.61, 95% CI 1.12–18.98; *p* = 0.03), early post-op heart rate (HR 0.96, 95% CI 0.93–0.99; *p* = 0.02), and CVP (HR 1.21, 95% CI 1.05–1.39; *p* = 0.01) as independent risk factors for RHF one-year following LVAD implantation (Table 3).

Multivariable Cox proportional cause-specific hazard regression analysis with RHF as the time-dependent covariate showed no association of RHF with increased risk of all-cause mortality at one-year after LVAD implantation (HR 1.002, 95% CI 0.99–1.01; *p* = 0.50) (Table 4). Covariates for this analysis were age, concomitant valve repair, INTERMACS profile, preoperative beta-blocker use as well as early post-op CVP/PCWP ratio, hematocrit, creatinine, blood urea nitrogen, total bilirubin, gamma glutamyl transferase, and albumin.

### 3.4. Pump Parameters

In HM3 patients, the pump speed setting (RHF: 5300 (350) rpm vs. no RHF: 5300 (700) rpm, *p* = 0.51), pump flow (RHF: 4.0 ± 0.6 lpm vs. no RHF: 4.2 ± 0.9 lpm, *p* = 0.65), and pulsatility index (RHF: 2.8 (0.8) vs. no RHF: 2.7 (1.5), *p* = 0.84) were comparable at the time of LVAD implantation, but HVAD patients with RHF had a significantly lower pump flow pulsatility (RHF: 2.0 (1.5) lpm vs. no RHF: 3.0 (1.6) lpm, *p* = 0.007) at similar speeds (RHF: 2450 (600) rpm vs. no RHF: 2500 (400) rpm, *p* = 0.51) and pump flows (RHF: 4.3 (1.7) lpm vs. no RHF: 4.3 (1.2) lpm, *p* = 0.98)

## 4. Discussion

The development of RHF after LVAD implantation remains a serious problem that needs to be addressed and has been studied extensively in the past to identify potentially modifiable risk factors.

The major finding of this study is the identification of the HVAD device as a significant risk factor for postoperative RHF compared with the HM3 device (Table 3). We also identified postoperative beta blocker administration and subsequent lower heart rate as an independent risk factor for RHF, consistent with previous studies on this topic [4,17]. Although it did not reach a statistical level of significance, a trend toward lower daily beta blocker doses were observed in patients with the HM3 compared to those with the HVAD device (3.75 mg vs. 5 mg; *p* = 0.085). We hypothesized that the results of previous clinical trials demonstrating the superiority of the HM3 device with respect to thromboembolic events [11,12,13,14] may have led to differences in the stringency of blood pressure management and thus lower doses of beta blockers in patients implanted with the HM3. Thus, our results suggest that it is not the device design per se, but differences in best practices in patient care that lead to a lower incidence of RHF in patients with the HM3 device. Although the HVAD has recently been withdrawn from the market by the manufacturer, the results of this study are still relevant as approximately 4000 patients worldwide remain on active support.

Beta-blocker therapy is an important tool in the treatment of HF because it reduces chronic activation of the sympathetic nervous system. It has been shown to significantly reduce mortality in HF patients [18] and is associated with a reduction in readmissions after LVAD implantation [19]. On the other hand, it has been demonstrated [20] that following beta blocker withdrawal, patients increased their cardiac output by 28% with no change in mean pulmonary artery pressure, resulting in a 19% decrease in pulmonary vascular resistance. The increases in cardiac output were related to a 25% increase in heart rate [20], thus these results implicate the importance of the right dose of beta blocker therapy and that the right balance to protect right heart function seems easier to maintain in patients with the HM3 device. Furthermore, our results clearly demonstrated that the occurrence of RHF was no risk factor for mortality in our cohort (Table 4), suggesting that development of late RHF was well manageable without a reduction in survival, at least in the first year after LVAD implantation. Our findings are in contrast to a previous published multicenter study [21] where LVAD patients suffering from late RHF had worse survival and a higher cumulative incidence of major adverse events. A possible reason for our divergent results could be the more pragmatic use of the tempRVAD at the time of LVAD implantation in patients with mild to moderate preoperative right ventricular failure, whereas Rame et al. [21] did not include such patients. It could be hypothesized that the perioperative use of tempRVADs has a beneficial effect on the development of late RHF. However, this possible effect needs to be further investigated in future studies.

Since the introduction of less invasive (LIS) LVAD implantation techniques using a combination of left thoracotomy and upper hemi-sternotomy instead of the conventional full sternotomy [22], it has been hypothesized that patients may also benefit in terms of RV protection. This hypothesis is based on the observation that longer time on CPB and increased peri- and postoperative bleeding are associated with RHF [4] and that the less invasive approach minimizes both of these factors [23]. Pasrija et al. [10] compared implantation methods in terms of their impact on RHF and observed a significantly lower incidence of severe RHF in patients with an LIS approach. In our cohort, no differences in implantation technique were found between patients with vs. without RHF. One possible explanation for this discrepancy is that the authors [10] distinguished between mild, moderate, and severe RHF and apparently found no difference in the overall population, which is consistent with our results.

Another interesting finding was the differences in pump parameters already at the time of implantation. Patients who developed RHF postoperatively and had a HVAD device implanted had significant lower pump flow pulsatility at comparable speed settings than patients without RHF (2.0 vs. 3.0, *p* = 0.007). This may reflect the already reduced right ventricular contractility in these patients at the time of LVAD implantation, making them vulnerable to postoperative RHF, and should be further investigated in the future. However, no differences in pump parameters were observed in patients receiving a HM3 device, which could be explained at least partially by the different pump characteristics between HVAD and HM3. Differences in patient characteristics should also be considered (Appendix A Table A2) as HVAD patients were significantly younger (59.0 vs. 64.5 years, *p* = 0.048), but represented the sicker patient cohort with a significantly higher proportion of INTERMACS level 1 and 2 compared to the HM3 cohort.

Finally, we identified an elevated early post-op CVP as independent “modifiable” risk factor for the development of RHF, which has already been described as preoperative risk factor pre-LVAD implantation [24,25]. Moreover, analysis of laboratory parameters showed a significantly higher pre-operative total bilirubin in patients who developed RHF postoperatively, which has also been previously described [17,26]. Thirty days after LVAD implantation, patients who later developed RHF had significantly reduced serum levels of hemoglobin and platelets, and elevated CRP compared to patients without RHF, possibly implicating a higher incidence of bleeding events in these patients, which is in line with the findings of Rame et al. [21], who reported an increased likelihood of gastrointestinal bleeding in patients with late RHF. Terzic et al. [27] recently found that proBNP and LDH levels may be useful prognostic indicators for the development of early RHF, which was not demonstrated for late RHF in our study.

Our study has limitations because data analysis was limited to available hemodynamic, clinical, and laboratory variables in medical records and the study cohort included only patients from a single center; therefore, it is not clear how reproducible our risk factors and biomarkers would be if applied to other institutions including different clinical judgment and best practices in RHF management. Another inherent problem in all studies published on this topic is that there remains no universally accepted definition and classification of RHF after LVAD implantation. Therefore, we tried to match our definition closely to that defined by other registries and studies [16,17,21] and refrained from analyzing echocardiographic data because the validity of isolated echocardiographic parameters should be taken with caution [28] because of the potential influence of high pulmonary vascular resistance, the presence of secondary tricuspid regurgitation, and dynamic changes in volume (over)load.

## 5. Conclusions

To conclude, HVAD compared to HM3 use, lower heart rate, and higher CVP in the early postoperative period were independent risk factors for RHF following LVAD implantation, but no effect of RHF on survival was detected.

## Figures and Tables

**Table 1:** Baseline demographics including intraoperative variables, comorbidities, and preoperative laboratory and hemodynamic parameters of the study population (n = 145). Abbreviations: BMI, body mass index; INTERMACS, Interagency Registry for Mechanically Assisted Circulatory Support; CPB, cardiopulmonary bypass; AVR, aortic valve repair; TVR, tricuspid valve repair ECLS, extracorporeal life support; BUN, blood urea nitrogen; Gamma GT, gamma glutamyl transferase; CRP, C-reactive protein; LDH, lactate dehydrogenase; proBNP, pro-brain natriuretic peptide; CK, creatine kinase; CVP, central venous pressure; PCWP, pulmonary capillary wedge pressure. Bold *p*-values denote statistical significance at *p* < 0.05.

Variable n (%), Median (IQR) or Mean ± SD	No RHF (n = 114)	RHF (n = 31)	*p*-Value
**Baseline characteristics**			
Sex (female)	16 (14.0)	4 (12.9)	1.00
Age at implant (years)	60.5 (13.0)	66.0 (20.0)	0.36
BMI (kg/m^2^)	26.1 (7.0)	27.6 (8.7)	0.36
INTERMACS level			
1	33 (29.0)	8 (25.8)	
2	23 (20.2)	7 (22.6)	0.83
3	22 (19.3)	5 (16.2)	
4–7	36 (31.5)	11 (35.4)	
Cardiomyopathy, ischemic	66 (58.9)	18 (58.1)	0.90
Strategy			
Destination therapy	35 (30.7)	14 (45.2)	
Bridge to transplantation	24 (21.1)	6 (19.4)	0.46
Bridge to candidacy	54 (47.4)	11 (35.5)	
Bridge to recovery	1 (0.9)	0 (0.0)	
**Intraoperative variables**			
Device			
HeartMate 3	61 (53.5)	9 (29.0)	**0.03**
HVAD	53 (46.5)	22 (71.0)	
Implantation technique, less invasive	40 (35.1)	14 (46.7)	0.29
Implantation with CPB	89 (78.8)	22 (71.0)	0.07
Concomitant temporary RVAD implantation	32 (28.1)	13 (41.9)	0.19
Concomitant valve surgery			
AVR	5 (4.4)	2 (6.5)	
TVR	13 (11.4)	4 (12.9)	
AVR + TVR	1 (0.9)	0 (0.0)	0.89
Other	8 (7.0)	1 (3.2)	
None	87 (76.3)	24 (77.4)	
**Comorbidities**			
Prae ECLS	28 (25.0)	7 (25.0)	1.00
Mechanical ventilation	14 (16.5)	1 (5.0)	0.29
Diabetes mellitus	34 (29.8)	10 (33.3)	0.82
Hypertension	46 (40.4)	9 (30.0)	0.40
Myocardial infarction	77 (67.5)	20 (66.7)	1.00
**Laboratory parameters**			
Hemoglobin (g/dL)	11.3 (4.2)	11.9 (3.6)	0.65
Hematocrit (%)	34.0 (11.0)	36.6 (9.9)	0.57
Platelets (g/L)	212.0 (124.0)	190.5 (135.3)	0.24
Leucocytes (g/L)	8.2 (3.7)	8.8 (5.2)	0.92
Serum creatinine (mg/dL)	1.3 (0.9)	1.5 (0.8)	0.31
BUN (mg/dL)	27.5 (23.2)	27.2 (20.3)	0.95
Total bilirubin (mg/dL)	1.0 (1.3)	1.5 (1.5)	**0.03**
Gamma GT (U/L)	98.0 (114.0)	72.0 (81.0)	0.06
CRP (mg/dL)	2.4 (4.4)	1.8 (3.1)	0.63
LDH (U/L)	244.0 (171.0)	251.0 (130.8)	0.65
proBNP (pg/mL)	9408.5 ± 7570.0	7687.1 ± 5817.4	0.45
Albumin (g/L)	33.9 (11.2)	34.5 (10.3)	0.49
CK (U/L)	66.5 (96.3)	68.0 (104.0)	0.78
**Hemodynamic parameters**			
CVP (mmHg)	14.0 (7.0)	16.5 (7.0)	**0.01**
PCWP (mmHg)	21.4 ± 7.4	22.0 ± 8.1	0.84
CVP/PCWP ratio	0.75 ± 0.3	0.83 ± 0.3	0.50
Systolic pulmonary artery pressure (mmHg)	42.0 ± 20.0	41.7 ± 15.8	0.94
Mean arterial blood pressure (mmHg)	71.5 (15.0)	73.0 (18.0)	0.62
Cardiac output (L/min)	3.5 (1.7)	3.5 (2.3)	0.86
Heart rate (bpm)	82.0 (35.0)	81.0 (35.0)	0.90

**Table 2:** Early post-op and 30-day laboratory and hemodynamic parameters of LVAD. Patients, stratified by occurrence of RHF. Abbreviations: BUN, blood urea nitrogen; Gamma GT, gamma glutamyl transferase; CRP, C-reactive protein; LDH, lactate dehydrogenase; proBNP, pro-brain natriuretic peptide; CK, creatine kinase; CVP, central venous pressure; PCWP, pulmonary capillary wedge pressure. Bold *p*-values denote statistical significance at *p* < 0.05.

Variable, n (%), Median (IQR) or Mean ± SD	No RHF (n = 114)	RHF (n = 31)	*p*-Value
**Early post-op**			
**Laboratory parameters**			
Hemoglobin (g/dL)	10.8 (1.6)	11.2 (1.5)	0.18
Hematocrit (%)	31.8 (5.4)	33.4 (3.9)	0.13
Platelets (g/L)	151.5 (101.5)	135.0 (93.5)	0.50
Leucocytes (g/L)	12.2 (6.7)	13.4 (7.4)	0.49
Serum creatinine (mg/dL)	1.3 (0.8)	1.4 (0.5)	0.19
BUN (mg/dL)	25.1 (18.8)	25.1 (13.1)	0.78
Total bilirubin (mg/dL)	1.8 (2.1)	2.8 (2.6)	0.21
Gamma GT (U/L)	52.0 (61.0)	39.0 (38.8)	0.06
CRP (mg/dL)	1.6 (3.2)	1.7 (3.3)	0.32
LDH (U/L)	331.0 (146.0)	386.0 (249.0)	0.25
proBNP (pg/mL)	4462.0 (4454.0)	5376.0 (3423.0)	0.92
Albumin (g/L)	27.6 ± 4.9	27.8 ± 5.0	0.81
CK (U/L)	414.0 (446.0)	369.0 (468.0)	0.64
**Hemodynamic parameters**			
CVP (mmHg)	13.0 (4.0)	14.0 (4.0)	**0.047**
PCWP (mmHg)	18.2 ± 7.6	19.9 ± 10.1	0.33
CVP/PCWP ratio	0.7 (0.5)	0.8 (0.5)	0.23
Systolic pulmonary artery pressure (mmHg)	43.0 (18.0)	42.0 (24.0)	0.86
Mean arterial blood pressure (mmHg)	73.0 (14.0)	72.0 (12.0)	0.83
Cardiac output (L/min)	5.3 (1.7)	4.8 (2.3)	0.17
Heart rate (bpm)	94.5 (21.0)	85.0 (32.0)	**0.003**
**30-days post-op**			
**Laboratory parameters**			
Hemoglobin (g/dL)	10.1 (2.1)	9.3 (1.6)	**0.01**
Hematocrit (%)	30.5 (6.2)	28.4 (5.3)	**0.03**
Platelets (g/L)	306.0 (168.0)	229.0 (205.0)	**0.01**
Leucocytes (g/L)	8.9 (4.3)	10.9 (8.6)	0.08
Serum creatinine (mg/dL)	0.9 (0.5)	1.0 (0.9)	0.27
BUN (mg/dL)	14.7 (14.6)	17.0 (17.7)	0.22
Total bilirubin (mg/dL)	0.6 (0.6)	0.8 (1.2)	**0.04**
Gamma GT (U/L)	117.0 (152.0)	97.0 (126.0)	0.79
CRP (mg/dL)	4.3 (5.1)	7.1 (6.2)	0.28
LDH (U/L)	282.0 (121.5)	338.0 (173.0)	0.18
proBNP (pg/mL)	2948.0 (6792.1)	-	-
Albumin (g/L)	31.8 ± 9.9	28.9 ± 5.8	0.08
CK (U/L)	35.0 (24.0)	30.0 (32.5)	0.34
**Hemodynamic parameters**			
CVP (mmHg)	10.0 (7.0)	12.0 (9.0)	0.39
PCWP (mmHg)	-	-	-
CVP/PCWP ratio	-	-	-
Systolic pulmonary artery pressure (mmHg)	-	-	-
Mean arterial blood pressure (mmHg)	87.0 (17.0)	82.0 (12.0)	0.83
Cardiac output (L/min)	-	-	-
Heart rate (bpm)	93.4 ± 14.4	85.8 ± 15.1	0.14

**Table 3:** Independent risk factors for the development of RHF one-year following LVAD implantation (multivariable Cox proportional hazard model). Abbreviations: RHF, right heart failure; LVAD, left ventricular assist device; CK, creatine kinase; CVP, central venous pressure; CRP, C-reactive protein. Bold *p*-values denote statistical significance at *p* < 0.05. Underlined factor was tested against reference category.

Variables	Hazard Ratio	Confidence Interval (95%)	*p*-Value
Beta blocker use preoperative (no vs. yes)	2.18	0.68–6.98	0.19
Hemoglobin preoperative (per unit)	0.29	0.06–1.59	0.16
CK preoperative (per unit)	1.00	0.99–1.01	0.56
Concomitant temporary RVAD support (no vs. yes)	1.76	0.39–8.02	0.47
Device (HeartMate 3 vs. HVAD)	4.61	1.12–18.98	**0.03**
Heart rate early post-op (per bpm)	0.96	0.93–0.99	**0.02**
CVP early post-op (per mmHg)	1.21	1.05–1.39	**0.01**
Hematocrit early post-op (per unit)	1.29	0.76–2.21	0.35
Platelets 30 d post-op (per unit)	0.99	0.99–1.00	0.25
Leukocytes 30 d post-op (per unit)	0.96	0.84–1.10	0.57
CRP 30 d post-op (per unit)	1.17	0.96–1.43	0.11
Total bilirubin 30 d post-op (per unit)	1.03	0.53–2.01	0.93
Albumin 30 d post-op (per unit)	1.05	0.93–1.19	0.40

**Table 4:** Survival during LVAD support (multivariable Cox proportional hazard model with RHF as a time-dependent covariate). Abbreviations: RHF, right heart failure; LVAD, left ventricular assist device; INTERMACS, Interagency Registry for Mechanically Assisted Circulatory Support; CVP, central venous pressure; PCWP, pulmonary artery capillary wedge pressure; BUN, blood urea nitrogen; Gamma GT, gamma glutamyl transferase. Underlined factor was tested against reference category.

Variables	Hazard Ratio	Confidence Interval (95%)	*p*-Value
RHF (no vs. yes)	1.00	0.99–1.01	0.49
Beta blocker use preoperative (no vs. yes)	1.46	0.54–3.92	0.46
Age (per year)	1.05	0.99–1.11	0.06
Concomitant valve surgery (no vs. yes)	2.84	0.98–8.26	0.06
INTERMACS profile			
1	Ref	Ref	0.13
2	0.98	0.31–3.13	0.97
3	0.22	0.04–1.26	0.09
4–7	0.26	0.05–1.26	0.09
CVP/PCWP ratio early post-op (per unit)	0.91	0.48–1.71	0.76
Hematocrit early post-op (per unit)	1.03	0.91–1.16	0.67
Creatinine early post-op (per unit)	1.44	0.79–2.62	0.23
BUN early post-op (per unit)	1.03	0.99–1.06	0.17
Total bilirubin early post-op (per unit)	1.19	0.97–1.47	0.09
Gamma GT early post-op (per unit)	1.00	0.99–1.00	0.51
Albumin early post-op (per unit)	0.96	0.87–1.06	0.45

## Data Availability

The data presented in this study are available on request from the corresponding author.

## Appendix A

**Table A1:** Univariable Cox proportional cause-specific hazard regression analysis for the development of RHF one-year following LVAD implantation. Abbreviations: BMI, body mass index; INTERMACS, Interagency Registry for Mechanically Assisted Circulatory Support; CPB, cardiopulmonary bypass; AVR, aortic valve repair; TVR, tricuspid valve repair; ECLS, extracorporeal life support; BUN, blood urea nitrogen; Gamma GT, gamma glutamyl transferase; CRP, C-reactive protein; LDH, lactate dehydrogenase; proBNP, pro-brain natriuretic peptide; CK, creatine kinase; CVP, central venous pressure; PCWP, pulmonary capillary wedge pressure. Bold *p*-values denote statistical significance at *p* < 0.05. Underlined factor was tested against reference category.

Variables	Hazard Ratio	Confidence Interval (95%)	*p*-Value
**Baseline demographics**			
**Baseline characteristics**			
Sex (female)	0.86	0.30–2.45	0.78
Age at implant	0.99	0.97–1.03	0.92
BMI (kg/m^2^)	1.03	0.97–1.11	0.33
INTERMACS level			0.83
1	ref	-	-
2	1.24	0.45–3.43	0.67
3	0.78	0.23–2.58	0.68
4–7	1.27	0.51–3.16	0.61
Cardiomyopathy (ischemic vs. dilative)	0.98	0.45–2.12	0.96
Strategy			0.56
Destination therapy	1.82	0.79–4.14	0.16
Bridge to transplantation	1.26	0.46–3.47	0.65
Bridge to candidacy	ref	-	-
Bridge to recovery	0.00	-	0.98
**Intraoperative variables**			
Device (HeartMate 3 vs. HVAD)	2.51	1.16–5.46	**0.02**
Implantation technique, less invasive	1.50	0.73–3.07	0.27
Implantation with CPB			
Concomitant temporary RVAD implantation	1.69	0.83–3.45	0.15
Concomitant valve surgery			0.95
AVR	1.31	0.31–5.53	0.72
TVR	1.17	0.41–3.37	0.77
AVR + TVR	0.00	-	0.98
Other	0.49	0.07–3.61	0.48
None	ref	-	-
**Comorbidities/medication**			
Prae ECLS	0.98	0.42–2.30	0.96
Mechanical ventilation	0.29	0.04–2.15	0.23
Diabetes mellitus	1.13	0.53–2.41	0.75
Hypertension	0.67	0.31–1.46	0.31
Myocardial infarction	1.08	0.51–2.32	0.84
Beta blocker use	1.83	0.79–4.20	0.16
**Laboratory parameters**			
Hemoglobin (g/dL)	1.20	0.92–1.57	0.18
Hematocrit (%)	1.01	0.96–1.06	0.65
Platelets (g/L)	0.99	0.99–1.00	0.23
Leucocytes (g/L)	1.02	0.93–1.10	0.72
Serum creatinine (mg/dL)	1.25	0.79–1.96	0.35
BUN (mg/dL)	1.00	0.98–1.02	0.97
Total bilirubin (mg/dL)	1.04	0.93–1.15	0.50
Gamma GT (U/L)	0.99	0.99–1.00	0.34
CRP (mg/dL)	0.98	0.91–1.05	0.53
LDH (U/L)	1.00	1.00–1.00	0.24
proBNP (pg/mL)	1.00	1.00–1.00	0.46
Albumin (g/L)	0.97	0.92–1.02	0.21
CK (U/L)	1.00	1.00–1.00	0.13
**Hemodynamic parameters**			
CVP (mmHg)	1.03	0.99–1.07	0.10
PCWP (mmHg)	1.01	0.91–1.11	0.86
CVP/PCWP ratio	2.50	0.98–31.59	0.48
Systolic pulmonary artery pressure (mmHg)	1.00	0.98–1.02	0.99
Mean arterial blood pressure (mmHg)	1.01	0.98–1.03	0.53
Cardiac output (L/min)	0.89	0.58–1.39	0.63
Heart rate (bpm)	0.99	0.98–1.01	0.96
**Early post-op**			
**Laboratory parameters**			
Hemoglobin (g/dL)	1.02	0.88–1.18	0.82
Hematocrit (%)	1.06	0.98–1.15	0.15
Platelets (g/L)	0.99	0.99–1.00	0.44
Leucocytes (g/L)	1.02	0.96–1.09	0.50
Serum creatinine (mg/dL)	1.13	0.72–1.77	0.61
BUN (mg/dL)	1.00	0.98–1.02	0.90
Total bilirubin (mg/dL)	1.03	0.90–1.18	0.70
Gamma GT (U/L)	0.99	0.99–1.00	0.48
CRP (mg/dL)	1.00	0.91–1.10	0.98
LDH (U/L)	1.00	1.00–1.00	0.81
proBNP (pg/mL)	1.00	1.00–1.00	0.82
Albumin (g/L)	1.01	0.94–1.09	0.81
CK (U/L)	1.00	1.00–1.00	0.20
**Hemodynamic parameters**			
CVP (mmHg)	1.11	1.03–1.19	**0.004**
PCWP (mmHg)	1.06	0.87–1.30	0.56
CVP/PCWP ratio	-	-	-
Systolic pulmonary artery pressure (mmHg)	1.01	0.98–1.04	0.37
Mean arterial blood pressure (mmHg)	1.00	0.97–1.03	0.94
Cardiac output (L/min)	0.85	0.59–1.21	0.37
Heart rate (bpm)	0.97	0.96–0.99	**0.001**
**30-days post-op**			
**Laboratory parameters**			
Hemoglobin (g/dL)	0.67	0.51–0.90	**0.01**
Hematocrit (%)	0.94	0.87–1.02	0.16
Platelets (g/L)	0.99	0.99–1.00	**0.02**
Leucocytes (g/L)	1.05	1.01–1.10	**0.02**
Serum creatinine (mg/dL)	1.11	0.82–1.49	0.51
BUN (mg/dL)	1.00	0.98–1.02	0.80
Total bilirubin (mg/dL)	1.13	1.03–1.24	**0.01**
Gamma GT (U/L)	1.00	0.99–1.00	0.95
CRP (mg/dL)	1.10	1.04–1.17	**0.001**
LDH (U/L)	1.00	0.99–1.00	0.97
proBNP (pg/mL)	1.00	0.99–1.00	0.70
Albumin (g/L)	0.94	0.89–1.00	0.07
CK (U/L)	0.99	0.98–1.01	0.29
**Hemodynamic parameters**			
CVP (mmHg)	0.99	0.94–1.06	0.89
PCWP (mmHg)	-	-	-
CVP/PCWP ratio	-	-	-
Systolic pulmonary artery pressure (mmHg)	-	-	-
Mean arterial blood pressure (mmHg)	0.98	0.95–1.01	0.23
Cardiac output (L/min)	-	-	-
Heart rate (bpm)	-	-	-

**Table A2:** Baseline demographics including intraoperative variables, comorbidities, and preoperative hemodynamic parameters stratified by device type (HeartMate 3 n = 70 and HVAD n = 75). Abbreviations: BMI, body mass index; INTERMACS, Interagency Registry for Mechanically Assisted Circulatory Support; CPB, cardiopulmonary bypass; RVAD, right ventricular assist device; AVR, aortic valve repair; TVR, tricuspid valve repair; ECLS, extracorporeal life support; CVP, central venous pressure; PCWP, pulmonary capillary wedge pressure. Bold p-values denote statistical significance at *p* < 0.05. Underlined factor was tested against reference category.

Variable n (%), Median (IQR) or Mean ± SD	HeartMate 3 (n = 70)	HVAD (n = 75)	*p*-Value
**Baseline characteristics**			
Sex (female)	7 (10.0)	13 (17.3)	0.23
Age at implant (years)	64.5 (13.0)	59.0 (15.0)	**0.048**
BMI (kg/m^2^)	27.1 (7.7)	25.4 (6.1)	0.14
INTERMACS level			
1	14 (20.0)	27 (37.0)	
2	13 (18.6)	17 (23.3)	**0.017**
3	12 (17.1)	14 (19.2)	
4–7	31 (44.3)	15 (20.5)	
Cardiomyopathy, ischemic	39 (55.7)	45 (61.6)	0.28
Strategy			
Destination therapy	26 (37.1)	22 (30.1)	
Bridge to transplantation	12 (17.1)	18 (24.7)	0.49
Bridge to candidacy	32 (45.7)	32 (43.8)	
Bridge to recovery	0 (0.0)	1 (1.4)	
**Intraoperative variables**			
Implantation technique, less invasive	19 (27.1)	35 (47.3)	**0.016**
Implantation with CPB	65 (94.2)	46 (61.3)	**<0.001**
Concomitant temporary RVAD implantation	22 (31.4)	23 (30.7)	0.99
Concomitant valve surgery			
AVR	4 (5.7)	3 (4.0)	
TVR	11 (15.7)	6 (8.0)	
AVR + TVR	0 (0.0)	1 (1.3)	0.52
Other	4 (5.7)	5 (6.7)	
None	51 (72.9)	60 (80.0)	
**Comorbidities**			
Prae ECLS	11 (16.4)	24 (32.9)	**0.032**
Mechanical ventilation	5 (9.3)	10 (19.6)	0.17
Diabetes mellitus	24 (34.3)	20 (27.0)	0.37
Hypertension	30 (42.9)	25 (33.8)	0.31
Myocardial infarction	50 (71.4)	47 (63.5)	0.38
**Hemodynamic parameters**			
CVP (mmHg)	14.0 (7.5)	15.0 (7.0)	0.15
PCWP (mmHg)	20.3 ± 7.1	22.4 ± 7.8	0.37
CVP/PCWP ratio	0.78 ± 0.3	0.75 ± 0.3	0.73
Systolic pulmonary artery pressure (mmHg)	44.5 ± 20.9	39.4 ± 16.9	0.12
Mean arterial blood pressure (mmHg)	69.5 (17.3)	73.0 (14.0)	0.40
Cardiac output (L/min)	3.4 (1.5)	3.6 (1.8)	0.67
Heart rate (bpm)	87.5 (39.0)	83.0 (28.0)	0.92
